# Emotion Understanding in Clinically Anxious Children: A Preliminary Investigation

**DOI:** 10.3389/fpsyg.2015.01916

**Published:** 2015-12-18

**Authors:** Patrick K. Bender, Francisco Pons, Paul L. Harris, Barbara H. Esbjørn, Marie L. Reinholdt-Dunne

**Affiliations:** ^1^Department of Psychology, University of CopenhagenCopenhagen, Denmark; ^2^Department of Psychology, University of OsloOslo, Norway; ^3^Graduate School of Education, Harvard University, CambridgeMA, USA

**Keywords:** emotion understanding, anxiety, emotion regulation, attachment, children, clinical sample

## Abstract

Children’s understanding of the nature, origins and consequences of emotions has been intensively investigated over the last 30–40 years. However, few empirical studies have looked at the relation between emotion understanding and anxiety in children and their results are mixed. The aim of the present study was to perform a preliminary investigation of the relationships between emotion understanding, anxiety, emotion dysregulation, and attachment security in clinically anxious children. A sample of 16 clinically anxious children (age 8–12, eight girls/boys) was assessed for emotion understanding (Test of Emotion Comprehension), anxiety (Screening for Child Anxiety Related Emotional Disorders-Revised and Anxiety Disorder Interview Schedule), emotion dysregulation (Difficulties in Emotion Regulation Scale) and attachment security (Security Scale). Children who reported more overall anxiety also reported greater difficulties in regulating their emotions, and were less securely attached to their parents. The results also showed that more specific symptoms of anxiety (i.e., OCD and PTSD) correlated not only with emotion dysregulation and attachment insecurity but also with emotion understanding. Finally, there were interrelations among emotion understanding, attachment security, and emotion dysregulation. The present results provide the first comprehensive evidence for a socio-emotional framework and its relevance to childhood anxiety.

## Introduction

Emotion understanding can be considered as the affective side of Theory-of-Mind (Wellman, 2014) and can be defined as the understanding of the nature, origins, consequences and regulation of emotion in the self and others (Harris et al., in press). Emotion understanding has been intensively investigated, especially in typically developing children, during the last 30–40 years. At least six conclusions have emerged from this large corpus of empirical investigations. First, in accordance with Piaget’s hypothesis about the development of consciousness “from the periphery to the center” (Piaget, 1974a,b), children’s understanding of emotions develops from a peripheral and superficial understanding of rather visible and non-reflective aspects of emotions (e.g., recognition of basic emotions, understanding of the impact of external causes and desires on emotions) to a more central and deeper understanding of the more invisible and reflective aspects of emotions (e.g., understanding of mixed and moral emotions, understanding of the possibility of regulating emotions by using cognitive strategies) via an intermediate “stage” where children understand the distinction between expressed and felt emotions, the impact of beliefs and memories on emotions and the impact of emotions on cognitions. Second, although some variation in rate of development has been observed across cultures (often related to socio-economic factors) this movement from the periphery to the center seems to be universal. Third, stable individual differences in children’s understanding of emotions have been observed from early childhood to late adolescence. Fourth, many interrelated social, cognitive and emotional factors such as children’s language, intelligence, executive functions, and maternal attachment relationships (including maternal sensitivity, as well as emotional responsiveness and communication) contribute to these developmental changes and individual differences. Fifth, children’s emotion understanding is related, not only to the quality of their psychological well-being (self-esteem, anger, behavioral problems, etc.) and their social relationships with peers and adults (friendship, popularity, cooperation, etc.) but also to their ability to resolve cognitive problems alone or in a group. Sixth, it is possible to help typical and challenged children to improve their emotion understanding via, for example, cognitive-behavioral programs, language-based interventions or philosophically based programs both in an experimental setting and at school (e.g., Marchetti et al., 2006; Gavazzi and Ornaghi, 2011; Nunez, 2011; Daniel and Gimenez-Dasi, 2012; Albanese and Molina, 2013; Andrés-Roqueta et al., 2013; Baron-Cohen et al., 2013; Molina et al., 2014; Harris et al., in press; Viana et al., submitted, for reviews and illustrations).

This corpus of investigations represents a substantial advance in our comprehension of children’s understanding of emotions. However, although several studies have looked at emotion understanding in challenged children (e.g., autistic, deaf, with specific language impairment), few have looked at the relation between emotion understanding and anxiety in children. Anxiety is an emotional disorder involving the experience of fear and danger that is either irrational and/or disproportionate to the perceived threat and has a negative impact on one or more areas of children’s normal functioning and/or psychosocial development (e.g., Fonseca and Perrin, 2011). Studies that have investigated the relation between children’s understanding of emotions and anxiety show mixed results. For example, studies have shown that children suffering from social anxiety have difficulties interpreting others’ facial (Simonian et al., 2001) and vocal (McClure and Nowicki, 2001) emotional cues. It has also been demonstrated that socially anxious children experience difficulties in understanding the relations between emotions, intentions, and beliefs in social situations (Banerjee and Henderson, 2001), and that a decreased ability to differentiate between emotions relates to social anxiety in children and adolescents (Rieffe et al., 2008). Sprung and Harris (2010) found that, in hurricane Katrina-exposed children, there was a positive correlation between their knowledge about thinking (including emotions) and their capacity to report on their negative intrusive thoughts. Southam-Gerow and Kendall (2000) found that anxious children have a less developed understanding of the possibility of hiding and changing emotions. A recent meta-analysis found a small-to-medium, negative correlation between general internalizing problems, such as anxiety and depression, and children’s ability to understand emotional cues (Trentacosta and Fine, 2010).

The relation between children’s understanding of emotions and their ability to regulate those emotions also deserves attention. The regulation of emotions is a complex and dynamic process involving the ability to assess the context surrounding an emotional experience, identifying and evaluating the emotional experience, as well as modifying the expression of emotion in accordance with personal goals and social demands (e.g., Jacob et al., 2011). It has been suggested that children must possess an understanding of emotions in order to be able to effectively regulate their emotions (Suveg et al., 2009; Izard et al., 2011). Indeed, in an intervention study, Izard et al. (2008) employed an emotion-based prevention program developed for young children, and showed that gains in emotion regulation were mediated by gains in emotion understanding. Other research has found that a better emotional understanding relates to improved emotion regulation abilities in children (Cunningham et al., 2009).

In a different line of research, the links between children’s emotion understanding and parent–child attachment relationships have been investigated. An attachment relationship may be understood as the bond between child and caregiver, which constitutes an emotional and behavioral system aimed at establishing and maintaining caregiver proximity in threatening situations (e.g., Manassis, 2011). According to Bowlby (1973, 1982), caregivers who respond sensitively and consistently to their children’s attachment-related needs and behaviors lay the foundation for the development of a secure attachment relationship. Securely attached children come to think of themselves as being able to elicit proximity and care from their attachment figures in times of distress, which allows them to explore their environment confident that they will be able to elicit help should they need it. Research investigating this association has shown that well-functioning attachment relations within families play an important role in the development of children’s emotional understanding (see e.g., de Rosnay and Harris, 2002; Ontai and Thompson, 2002; de Rosnay et al., 2008). For example, Steele et al. (1999) found that infant-mother attachment at 1 year predicted children understanding of mixed emotions 5 years later.

In line with the research concerning children’s emotion understanding, research focusing on childhood anxiety has also directed its attention toward the relation between anxiety disorders and insecure attachment relationships with parents (e.g., Muris et al., 2000, 2001; Shamir-Essakow et al., 2005), as well as difficulties in regulating emotions (e.g., Suveg and Zeman, 2004; Carthy et al., 2010; Neumann et al., 2010). Although several authors have argued that emotion regulation abilities in children may be related to attachment security (e.g., Cassidy, 1994; Sroufe, 1996), the vast majority of studies has investigated either the association between anxiety and attachment security, or the association between anxiety and emotion regulation abilities (e.g., Esbjørn et al., 2012, for a review). Very few studies have investigated the relation between emotion regulation, attachment security, and anxiety within the same individuals (however, see Bosquet and Egeland, 2006; Brumariu et al., 2012; Bender et al., 2015, for exceptions). Furthermore, many of these studies have been conducted using community samples. Thus, an examination of the associations among anxiety, emotion regulation, and attachment security within clinical populations is called for in order to see whether the interrelations found in community samples correspond to those found in clinical populations (Esbjørn et al., 2012).

In summary, to the best of our knowledge, no research has simultaneously examined the relationships between emotion understanding, anxiety, emotion regulation and attachment security either within the same individuals or in clinically anxious children. By investigating the relation among these four variables, we should not only expand the field of research on the development of emotion understanding among atypical children (e.g., beyond autism) but also improve our understanding of the interplay among these variables. It is also important to combine these variables in the same study so as to expand our knowledge about the role of socio-emotional factors in the etiology and maintenance of anxiety disorders in childhood. Although research has demonstrated that traditional cognitive-behavioral therapy (CBT) is an effective tool in relation to childhood anxiety (e.g., Barrett et al., 2001; Hirshfeld-Becker et al., 2008), meta-analytical investigations have shown only moderate effect sizes for the efficacy of CBT with children and called for the improvement of traditional CBT treatment (Reynolds et al., 2012). In order to develop treatment protocols that target a greater range of the factors involved in the etiology and maintenance of childhood anxiety, and thus, maximize treatment outcome, a simultaneous investigation of the various factors related to childhood anxiety disorders, such as emotion understanding, emotion regulation and attachment, is called for.

The goal of the present study was to conduct a preliminary examination of the associations between children’s anxiety, their understanding of emotions, their difficulties regulating emotions, and the quality of parent–child attachment relationships in a sample of children diagnosed with anxiety disorders. Because many of the previous investigations have been conducted using community samples, we asked whether we would find the same associations among anxiety, emotion understanding, emotion regulation, and attachment security, as other studies; namely that more anxious children will show more limited emotion understanding, greater difficulties regulating their emotions, and report less attachment security than less anxious children (e.g., Brumariu et al., 2012; Bender et al., 2015). Additionally, we asked whether a better emotional understanding in children would be associated with less anxiety, fewer emotion regulation difficulties, as well as more secure attachment relationships with parents, as suggested, in theory, by the literature.

## Materials and Methods

### Participants

Participants were 16 children (eight girls, eight boys) ranging from 8 to 12 years (*M* = 10.38, *SD* = 1.54). Children had been referred for anxiety treatment to the Copenhagen Child Anxiety Project (CCAP) at the University of Copenhagen; they were a sub-sample of children referred for participation in a randomized trial, assessing the efficacy of two types of CBT intervention for anxiety (Esbjørn et al., 2015). For intervention purposes, all children had to have one of four anxiety disorders [generalized anxiety disorder (GAD), separation anxiety disorder (SAD), specific phobia (SP) and/or social phobia (SoP)] as their primary diagnosis. Children were diagnosed using the ADIS-IV interview assessment; only children scoring above the clinical cut-off for at least one of the anxiety disorders were included. Because studies have shown that children with cognitive developmental delays exhibit poorer social skills and problem solving abilities than typically developing children (Fenning et al., 2011; Wieland et al., 2014), only children with a full-scale IQ of 85–115 were examined in the current study (mean IQ for the sample was 105.13; *SD* = 8.29). Also, because of previously found gender differences with regard to childhood anxiety and emotion regulation difficulties (e.g., Bender et al., 2012), we balanced gender in the current study by including an equal number of boys and girls. From the eligible sample of 21 children (13 girls, 8 boys), five girls were excluded (the eight girls included in the current study were matched as closely as possible to boys with respect to IQ and age). All children were Danish and had provided valid answers to all measures of interest at pre-treatment. See **Table 1** for additional clinical and demographic information.

**Table 1:** Sample clinical and demographic information.

	*n*
Primary ADIS-IV diagnosis (*n* = 16)	
Generalized anxiety disorder	3
Separation anxiety disorder	7
Social phobia	1
Specific phobia	5
Number of ADIS-IV diagnoses (*n* = 16)	
One diagnosis	5
Two diagnoses	7
Three diagnoses	4
Family status (*n* = 16)	
Child lives with both parents	12
Parents are divorced	4
Number of siblings (n = 16)	
Three siblings	3
Two siblings	2
One sibling	9
No siblings	2
Educational level mother (*n* = 14)	
Short	2
Medium (vocational; business, technical)	1
Medium (academic; BA)	3
Long (Master’s)	8
Educational level father (*n* = 13)	
Medium (vocational; business, technical)	3
Medium (academic; BA)	2
Long (Master’s)	8
Family income (*n* = 13)	
Below average	1
Average	1
Above average	3
High	8

*See http://www.dst.dk/en/Statistik/emner/indkomster/familieindkomster for the Danish average family income.*

### Procedure

All data presented here constitute pre-treatment data. Children were assessed at the University Clinic at the University of Copenhagen prior to their participation in the CBT programs. All testing was carried out on the same day by members of the CCAP staff and students associated with CCAP. Prior to inclusion in the project, parents had provided informed, written consent to participate in the study. On the day of testing, children and parents were informed that they could terminate participation in the study at any time. Parents and children were interviewed and assessed separately. For child questionnaire measures, experimenters read aloud the various items and children were asked to check the answers they felt were most appropriate.

### Measures

#### Anxiety Disorders Interview Schedule for DSM-IV: Child and Parent Versions (ADIS-IV-C/P)

The ADIS-IV-C/P (Silverman and Albano, 1996) is a semi-structured interview assessing anxiety disorders and other types of psychopathology in children according to DSM-IV criteria (American Psychiatric Association [APA], 1994). The instrument consists of two separate interviews, one conducted with the child, and one conducted with the child’s parents. Both children and parents rate the severity of symptoms experienced by the child on a scale from 0 to 8, where a rating of 4 or higher indicates clinical levels of difficulties and leads to a diagnosis of the disorder in question. Based on the separate child and parent interviews, a combined diagnostic description of the child is created, summarizing any number of diagnoses obtained via both child and parent severity ratings. In the current study, most children presented with one or two anxiety disorders with a maximum of three diagnoses recorded per child (see **Table 1**). The ADIS-IV-C/P has been shown to be a reliable instrument for deriving DSM-IV anxiety disorder diagnoses in children (Silverman et al., 2001).

#### Screen for Child Anxiety Related Emotional Disorders-Revised (SCARED-R)

The SCARED-R (Muris et al., 1998; Muris and Steerneman, 2001) is a self-report questionnaire, assessing DSM-IV related anxiety disorder symptoms in children. The SCARED-R consists of 66 items and nine subscales, which provide indices of the following DSM-IV anxiety disorders: (1) separation anxiety disorder (SAD), (2) panic disorder, (3) social phobia, (4) obsessive-compulsive disorder (OCD), (5) post-traumatic stress disorder (PTSD), (6) generalized anxiety disorder (GAD), and (7) specific phobias. Items are rated on a 3-point Likert scale (0 = almost never; 1 = sometimes, and 2 = often) and assess the frequency with which children experience the symptoms described by the various items. The total score of the SCARED-R is reported as a sum score ranging from 0 to 132. The SCARED-R has shown good internal consistency (Muris and Steerneman, 2001). In this study, Cronbach’s α was 0.93 for the SCARED-R total score; subscale’s α ranged from 0.62 to 0.90 (two items of the OCD subscale had zero variance and were removed from the Cronbach’s α calculations).

#### Test of Emotion Comprehension (TEC)

The TEC (Pons and Harris, 2000) consists of a picture book containing cartoon scenarios, which are accompanied by various descriptions and stories designed to test children’s understanding of emotions. Each scenario comes with four possible emotional story outcomes, represented as the facial expressions of the story protagonist, which are left blank in the scenario itself. After children are introduced to the individual scenario and the experimenter has read the accompanying story, children are asked to attribute an emotion to the story protagonist(s) by pointing at the most appropriate of the four possible emotional outcomes. The TEC assesses nine different components of emotion understanding: (1) recognition of facial expressions, (2) understanding of external causes of emotions, (3) understanding of desire-based emotions, (4) understanding of belief-based emotions, (5) understanding of the influence of a reminder on present emotional states, (6) understanding of the possibility to regulate emotional states, (7) understanding of the possibility of hiding emotional states, (8) understanding of mixed emotions, and (9) understanding of moral emotions. The TEC yields a total score from 0 to 9 based on children’s overall level of emotion understanding. The TEC has been translated into 23 languages until now. It has shown good test–retest reliability, as well as concurrent, criterion and construct validity. It has been standardized in Italian and Portuguese (see e.g., Pons et al., 2014 for a recent review).

#### Difficulties in Emotion Regulation Scale (DERS)

The DERS (Gratz and Roemer, 2004) is a self-report questionnaire, which consists of 36 items and measures difficulties with regard to emotion regulation. Items are rated on a 5-point Likert scale, ranging from 1 (almost never) to 5 (almost always) and assess the frequency with which respondents experience emotion regulation difficulties. The DERS consists of six subscales: (1) non-acceptance of negative emotional responses, (2) difficulties engaging in goal-directed behavior when experiencing negative emotions, (3) difficulties controlling impulses when experiencing negative emotions, (4) lack of awareness of emotional responses, (5) limited access to emotion regulation strategies perceived as effective, and (6) lack of clarity of emotional responses. The DERS has shown good internal consistency (Neumann et al., 2010). However, it has been suggested that the subscale assessing lack of awareness of emotional responses be removed when interpreting the DERS total score (Bardeen et al., 2012). In the current study, the lack of awareness subscale was removed and the DERS index computed based on the remaining five subscales, yielding a score from 1 to 5. Cronbach’s α for the DERS total without the awareness subscale (items *n* = 30) was 0.92.

#### Security Scale

The Security Scale (Kerns et al., 1996) is a self-report measure, which assesses children’s perceptions of security in parent–child relationships. The instrument consists of 15 items, which assess: (1) the degree to which children perceive their parents as being responsive and available, (2) children’s tendency to rely on their parents in times of distress, and (3) children’s ease and interest in communicating with their parents. Items are rated on a 4-point scale using an opposing statements format (“Some kids... Other kids...”). For example, “Some kids find it easy to trust their mom BUT other kids are not sure if they can trust their mom.” Children are asked to indicate which of the two statements is more characteristic of them, and whether this statement is “really true” or “sort of true” for them. The Security Scale yields an overall score from 1 to 4, where higher scores indicate a more secure attachment. In order to get a more comprehensive picture of children’s attachment relationships, children were asked to fill out the Security Scale for both their mothers and fathers. These two scores were then added and divided by two to get an overall parental attachment security score, ranging from 1 to 4. The Security Scale has shown good internal consistency (Kerns et al., 1996; Van Ryzin and Leve, 2012). In this study, Cronbach’s α was 0.81 for mother, and 0.73 for father.

#### Cognitive Ability/IQ Index: Wechsler Intelligence Scale for Children-III (WISC-III) Subtests

In the present study, an index of children’s IQ was obtained using the WISC-III (Wechsler, 1991), which assesses children’s verbal, performance, and full scale IQ. The subtests employed in the current study included the verbal subtests Information, Similarities, Vocabulary, and Arithmetic, as well as the performance subtests Picture arrangement, Picture Completion, Coding, and Block Design. Based on children’s scores on these subtests, the full scale IQ was computed.

## Results

### Data Analyses

In this study, we employed one-sample *t*-tests to test for differences between the various measure mean scores obtained here and mean scores found in other studies. One-sample *t*-tests compare a sample mean to a known or hypothesized value of the mean in the population. In this case, the hypothesized population means for the individual measures were the weighted overall means extracted from the literature (see Appendix A). To test for associations among the various measures, we used one-tailed, bivariate Pearson’s correlations, which measure the strength and direction, as well as the significance of linear relationships between pairs of variables. We chose to employ one-tailed correlations due to the small sample size of this preliminary study, as well as the directional assumptions regarding the correlation (i.e., positive versus negative) among the assessed variables.

In order to see whether the current sample of clinically anxious children differed from other samples of both non-clinical, as well as clinically anxious children, we first compared the mean scores obtained in this study to mean scores obtained from the literature. Next, we examined the relations among the overall measures of anxiety, emotion understanding, emotion regulation, and attachment security, to see whether we would find the associations that have been found in various, separate lines of research in this sample of clinically anxious children. Finally, we investigated the associations between the subscales of the SCARED-R and the overall measures of emotion understanding, emotion regulation, and attachment security, in order to see which aspects of children’s anxiety symptoms explained the relations among the overall measures. **Table 2** shows the sample means and correlations among the variables examined in this study.

**Table 2:** Sample means (*SD*) and correlations table.

		1	2	3	4	5	6	7	8	9	10	11
	*M* (*SD*)											

(1) TEC	7.81(1.33)	1										
(2) DERS	2.04(0.55)	-0.46^∗^	1									
(3) SecScale	3.29(0.39)	0.42^∗^	-0.42^∗^	1								
(4) ADIS	1.94(0.77)	-0.01	0.32	-0.20	1							
(5) SCARED-R	27.31(16.37)	-0.22	0.49^∗^	-0.49^∗^	0.71^∗∗^	1						
(6) SAD	0.48(0.28)	0.17	0.33	-0.24	0.71^∗∗^	0.72^∗∗^	1					
(7) Panic	0.29(0.28)	-0.13	0.37	-0.46^∗^	0.35	0.78^∗∗^	0.30	1				
(8) Soc. phobia	0.39(0.41)	-0.16	0.26	-0.34	0.67^∗∗^	0.83^∗∗^	0.59^∗∗^	0.48^∗^	1			
(9) OCD	0.31(0.26)	-0.51^∗^	0.62^∗∗^	-0.61^∗∗^	0.40	0.79^∗∗^	0.45^∗^	0.62^∗∗^	0.65^∗∗^	1		
(10) PTSD	0.44(0.51)	-0.63 ^∗∗^	0.41	-0.55^∗^	0.54^∗^	0.67^∗∗^	0.36	0.46^∗^	0.50^∗^	0.78^∗∗^	1	
(11) GAD	0.44(0.49)	-0.17	0.34	-0.34	0.80^∗∗^	0.85^∗∗^	0.67^∗∗^	0.45^∗^	0.78^∗∗^	0.57^∗^	0.62^∗∗^	1
(12) Spec. phobia	0.51(0.25)	-0.04	0.36	-0.24	0.36	0.76^∗∗^	0.42^∗^	0.79^∗∗^	0.59^∗∗^	0.46^∗^	0.16	0.45^∗^

*TEC, Test of Emotion Comprehension; DERS, Difficulties in Emotion Regulation Scale; SecScale, Security Scale (parents); ADIS, Anxiety Disorder Interview Schedule (number of diagnoses); SCARED-R, Screen for Child Anxiety Related Emotional Disorders-Revised. SCARED-R subscales (reported as mean values, range: 0–2): SAD, separation anxiety disorder; Panic, panic disorder; Soc. phobia, social phobia; OCD, obsessive-compulsive disorder; PTSD, post-traumatic stress disorder; GAD, generalized anxiety disorder; Spec. phobia, specific phobia. ^∗^*p* ≤ 0.05; ^∗∗^p < 0.01.*

#### Comparison with Clinically Anxious and Non-clinical Samples

In order to compare the mean scores obtained in this study to overall mean scores from the literature, one-sample *t*-tests were used (see Appendix A for a list of studies and values from the literature). Results showed that children in this study did not differ from community samples of children with regard to their level of anxiety (*M* = 27.31, *SD* = 16.37) and scored significantly lower than other samples of clinically anxious children from the US [*t*(15) = -6.00, *p* < 0.001]. However, results also showed that children who reported higher levels of anxiety also received a higher number of anxiety diagnoses (*r* = 0.71, *p* = 0.001). Results further showed that children in this study did not differ from community samples of children with regard to their emotion understanding (*M* = 7.81, *SD* = 1.33), their emotion regulation difficulties (*M* = 2.27, *SD* = 0.46), or their perceptions of parent–child attachment security (*M* = 3.29, *SD* = 0.39).

#### Relation between Anxiety, Emotion Regulation, Attachment and Emotion Understanding

One-tailed, bivariate Pearson’s correlations were used to investigate the relations among the overall measures of anxiety, emotion understanding, emotion regulation, and attachment security (see **Table 2**). Results showed that children who reported higher levels of anxiety had more difficulties regulating their emotions (*r* = 0.49, *p* = 0.03) and thought of their attachment relationships as less secure (*r* = -0.49, *p* = 0.03). Although negative, the relation between children’s overall level of anxiety and their level of emotion understanding was not significant. Correlation analyses also showed that children who had a more developed understanding of emotions had fewer difficulties regulating their emotions (*r* = -0.46, *p* = 0.04), and thought of their attachment relationships as more secure (*r* = 0.42, *p* = 0.05). Finally, analyses showed that children who reported perceptions of a more secure attachment relationship with their parents also reported fewer difficulties in regulating their emotions (*r* = -0.42, *p* = 0.05).

To determine which subscales of the SCARED-R accounted for the relations among the total SCARED-R scores and the other measures, one-tailed, bivariate Pearson’s correlations were conducted (see **Table 2**). The analyses showed that the association between anxiety and emotion regulation was largely due to an association between the OCD subscale and the DERS (*r* = 0.62, *p* = 0.005). The relation between anxiety and perceived attachment security was due to associations between the Security Scale and the Panic disorder subscale (*r* = -0.46, *p* = 0.04), the OCD subscale (*r* = -0.61, *p* = 0.007), and the PTSD subscale (*r* = -0.55, *p* = 0.01), respectively. Although the overall measures of anxiety and emotion understanding did not correlate significantly with each other, the subscale analyses showed significant associations between the TEC and the OCD subscale (*r* = -0.51, *p* = 0.02), as well as the PTSD subscale (*r* = -0.63, *p* = 0.004).

## Discussion

This was the first study to investigate the relations among anxiety, emotion understanding, emotion regulation, and attachment security in the same sample of clinically anxious children. The findings of the current study are in line with previous studies showing that more anxious children have greater difficulties in regulating their emotions (e.g., Carthy et al., 2010), and experience attachment relationships with their parents as less secure (e.g., Colonnesi et al., 2011). Also in line with previous findings, this study shows that a better emotional understanding in childhood is associated with higher attachment security (e.g., de Rosnay and Harris, 2002) and fewer emotion regulation difficulties (e.g., Izard et al., 2008). Finally, this study shows that children who report a more secure attachment also report fewer difficulties regulating their emotions. Although previous research has demonstrated these associations in separate studies, using predominantly community samples, the current study combines and extends these findings to children with anxiety disorders.

When investigating the relations between the subscales of the SCARED-R and the overall measures of emotion understanding, emotion regulation difficulties, and attachment security, respectively, results showed that the OCD subscale correlated positively with emotion dysregulation and negatively with emotion understanding and attachment security. The PTSD subscale correlated negatively with emotion understanding and attachment security. And finally, the Panic disorder subscale correlated negatively with attachment security. Although none of the children in this study were specifically diagnosed with OCD, PTSD, or Panic disorder, these subscales contain items that may also be regarded as more generic indices of intrusive anxiety problems (e.g., “I have thoughts that frighten me,” “I do things to get less scared of my thoughts,” “When frightened, I sweat a lot,” and “When frightened, my heart beats fast”). This study replicates and extends the findings of previous research on intrusive thoughts in hurricane-Katrina exposed children (Sprung and Harris, 2010); a better understanding of emotion is associated with less PTSD and OCD.

Although the present study cannot answer questions regarding the causal pathways connecting anxiety, emotion understanding, emotion dysregulation, and attachment security, the results obtained here once again underline the relevance of socio-emotional factors in relation to childhood anxiety. Based on theoretical conceptions and previous research, as well as the results obtained in this study, we can formulate testable hypotheses regarding the interactions among these factors. For example, research on the development of emotion understanding has shown that parents who establish secure attachment relationships with their children also tend to use more mental state language in conversation with their children (e.g., McQuaid et al., 2008). This is thought to not only provide a safe environment for children to experience an adequate range of their own emotions but also to teach children about emotions, their antecedents and consequences, via discussions with their parents (see Harris, 1999). Similarly, it has been argued that the defining characteristic of a secure attachment relationship is the effective parent-child co-regulation of children’s emotions in times of distress (Sroufe, 1996). This is thought to help children develop adequate strategies for handling their own emotional arousal and lay the basis for later emotional self-regulatory abilities (see Sroufe, 2005). Thus, one hypothesis is that children’s attachment relationships with parents may constitute the basis on which developing emotional competencies are built.

Consistent with previous findings, this study found a relation between children’s perceptions of attachment security and reported levels of anxiety. However, recent studies (e.g., Brumariu et al., 2012; Bender et al., 2015) raise questions regarding the nature of this association; that is whether attachment security relates directly to anxiety or whether the effect is mediated via other factors. Although this could not be tested in the current study, given the theoretical importance of attachment security to child emotional functioning (e.g., Cassidy, 1994), as well as the well-established link between emotional dysregulation and childhood anxiety, another hypothesis is that attachment security relates to anxiety via children’s emotional capacities, including children’s emotion understanding and regulation. Indeed, research suggests that the relation between attachment security and childhood anxiety may be mediated by children’s emotion regulation abilities (Brumariu et al., 2012; Bender et al., 2015).

Although some studies have found an association between children’s understanding of emotions and anxiety, there was no significant relation between the overall measures of these two factors in the current study (the only significant relation was between emotion understanding and the specific measures of PTSD and OCD). Children in this study were relatively old and scored high on emotion understanding. It is possible that emotion understanding is more strongly related to anxiety in younger children who have a less developed understanding of emotions. In any case, emotional understanding appears to be a part of the socio-emotional framework surrounding child anxiety via its links to attachment security and emotion dysregulation. However, whereas the association between emotion understanding and attachment security seems to be relatively clear, the relation between children’s understanding of emotions and their ability to regulate their own emotions is less clear. Although it is plausible that some understanding of emotions is necessary for an effective regulation of emotional states to take place, emotional regulation often precedes an explicit knowledge of emotions (Southam-Gerow and Kendall, 2002). More research is needed to study the relation between emotion understanding and emotion regulation in children with anxiety disorders. Meantime, we hypothesize a dynamic relationship between the two factors based on the assumption that the affective experiences that children collect via their emotion regulation efforts will influence the cognitive structures and processes related to the regulated emotional states, and vice versa (Rieffe et al., 2005; Pons et al., 2010).

**FIGURE 1:**
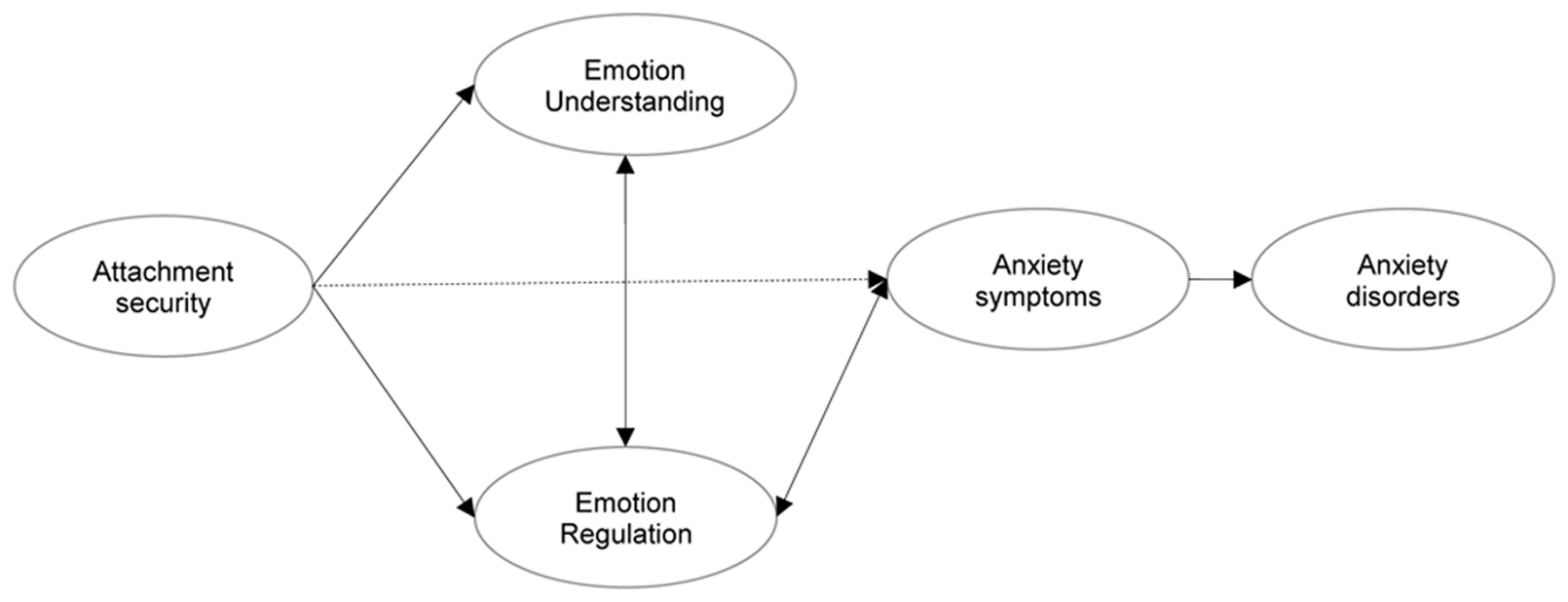
**Testable hypotheses regarding the socio-emotional framework of child anxiety**.

Taken together, the findings presented here and other findings and concepts found in the literature point to a series of testable hypotheses regarding the socio-emotional framework relevant to childhood anxiety (see **Figure 1**). The proposed framework is to be regarded as the beginnings of a conceptualization of the relevant socio-emotional factors and their relation to each other, as well as to child anxiety. Future research will need to refine and revise the hypotheses laid out here. Also, a number of other factors, such as behavioral inhibition (e.g., Shamir-Essakow et al., 2005), peer relations (e.g., Bosquet and Egeland, 2006), cognitive biases (see Hadwin et al., 2006), cognitive development (e.g., Fenning et al., 2011), and gender (e.g., Bender et al., 2012), are likely to be related to this framework, and future investigations need to examine how the various factors are associated with each other. It is our hope that the revision and development of the socio-emotional framework proposed here will lead to a better understanding of the etiology and maintenance of anxiety disorders in childhood, which in turn will help develop more effective prevention and treatment protocols for child anxiety.

An unexpected finding of the current study was that children showed lower levels of anxiety than clinically anxious children in other studies and did not differ from community samples of children with regard to their level of anxiety. However, previous research has found that Danish children tend to score lower on self-report measures assessing anxiety and worry than, for example, US children (e.g., Reinholdt-Dunne et al., 2012; Esbjørn et al., 2013), and that Danish individuals tend to overrate their own health in self-report measures (Jürges, 2007). Further, we have to keep in mind that community samples also include children who experience clinical levels of anxiety, and thus, anxiety levels found in community samples are not synonymous with the expected levels of anxiety found in positively non-anxious children. While we cannot definitely identify the reason for this finding, the high correlation (0.71) between children’s anxiety symptoms as assessed by the SCARED and the number of children’s ADIS-IV anxiety diagnoses indicates that we successfully assessed differentiating levels of anxiety in the current sample of Danish children diagnosed with anxiety disorders.

The present results cannot in themselves answer questions regarding causal pathways among the various factors. Furthermore, the findings presented here are preliminary and based on a small sample of clinically anxious children and should therefore be interpreted with caution. However, in an effort to control for possible confounding influences, the sample was carefully selected with respect to gender, age, as well as cognitive abilities. In sum, the results underline the links between anxiety, emotion understanding, emotion dysregulation, and attachment security, and highlight the importance of combining the various lines of research concerned with these factors. Although studies separately examining the individual associations have identified the factors of interest in relation to childhood anxiety, it is now time to develop a comprehensive picture of the interrelations among these factors, and future research should investigate these using larger samples, as well as longitudinal and experimental research designs. Based on this, as well as other studies and theoretical concepts, a socio-emotional framework was proposed, outlining hypotheses regarding the pathways connecting the various socio-emotional factors to each other, as well as anxiety in children.

## Conflict of Interest Statement

The authors declare that the research was conducted in the absence of any commercial or financial relationships that could be construed as a potential conflict of interest.
