# Supplementary material for: Emotion Understanding in Clinically Anxious Children: A Preliminary Investigation
Source: Front Psychol. 2015 Dec 18;6:1916. doi: 10.3389/fpsyg.2015.01916 (PMC4683184; doi:10.3389/fpsyg.2015.01916)
Supplement: Supplementary file 1 [file Data_Sheet_1.DOCX]

Appendix A

A search of the literature yielded several community studies with children of a comparable age range that included one or more of the measures employed in the current study and reported mean values for these measures. In the case of the SCARED-R, the search also yielded studies investigating clinically anxious children. A weighted overall mean was calculated for each measure using the means and sample sizes reported in the various studies. In order to compare the means obtained in the present study to these overall weighted means, one-sample *t*-tests were used, which compare a single sample mean to a specified constant, i.e., the weighted measure mean (see Results section).

| **Study** | **Measure** | **Sample** | **N** | **Age range** | **Mean** | **Weighted Mean** |
| --- | --- | --- | --- | --- | --- | --- |
| Bender et al. (2012) | SCARED-R | Community | 544 | 9-16 | 30.36 |  |
| Study 1; Muris, Merckelbach, Mayer, et al. (1998) | SCARED-R | Community | 75 | 9-12 | 22.80 |  |
| Study 2; Muris, Merckelbach, Mayer, et al. (1998) | SCARED-R | Community | 120 | 8-13 | 31.70 |  |
| Muris, Merckelbach, Schmidt, et al. (1998) | SCARED-R | Community | 674 | 8-13 | 30.43 |  |
| Muris, Merckelbach, et al. (2001) | SCARED-R | Community | 534 | 7-14 | 39.40 |  |
| Overall | SCARED-R | Community | 1947 | 7-16 |  | 32.66 |
| Muris, Dreessen, Bögels, Weckx, and van Melick (2004) | SCARED-R | Clinical | 81 | 7-17 | 51.20 |  |
| Muris, Mayer, Bartelds, Tierney, and Bogie (2001) | SCARED-R | Clinical | 36 | 8-13 | 69.30 |  |
| Muris and Steerneman (2001) | SCARED-R | Clinical | 25 | 8-17 | 50.30 |  |
| Overall | SCARED-R | Clinical | 142 | 7-17 |  | 55.63 |
| Pons, Lawson, Harris, and de Rosnay (2003) | TEC | Community | 40 | 8-11 | 7.23 |  |
| Pons and Harris (2005) | TEC | Community | 42 | 8-12 | 7.74 |  |
| Morra, Parrella, and Camba (2011) | TEC | Community | 41 | Grade 4-5 (mean ages: 10.9 and 11.8, respectively) | 7.24 |  |
| Albanese, De Stasio, Di Chiacchio, Fiorilli, and Pons (2010) | TEC | Community | 111 | 9-11 | 7.60 |  |
| Lecce, Zocchi, Pagnin, Palladino, and Taumoepeau (2010) | TEC | Community | 71 | 9-11 | 7.23 |  |
| Overall | TEC | Community | 305 | 8-12 |  | 7.44 |
| Vasilev, Crowell, Beauchaine, Mead, and Gatzke-Kopp (2009) | DERS | Community | 69 | 11-15 | 1.90 |  |
| Bender et al. (2012) | DERS | Community | 544 | 9-16 | 2.38 |  |
| Overall | DERS | Community | 613 | 9-16 |  | 2.33 |
| Kerns, Aspelmeier, Gentzler, and Grabill (2001) | Security Scale | Community | 171 | Grade 3 and 6 (mean ages: 9.12 and 12.08, respectively) | 3.37 |  |
| Cassidy, Aikins, and Chernoff (2003) | Security Scale | Community | 83 | 8-10 | 3.44 |  |
| Kerns, Tomich, Aspelmeier, and Contreras (2000) | Security Scale | Community | 176 | 8-13 | 3.37 |  |
| Study 1; Kerns et al. (1996) | Security Scale | Community | 74 | Grade 5 | 3.24 |  |
| Study 2; Kerns et al. (1996) | Security Scale | Community | 88 | 9-13 | 3.16 |  |
| Contreras, Kerns, Weimer, Gentzler, and Tomich (2000) | Security Scale | Community | 62 | 9-12 | 3.41 |  |
| Overall | Security Scale | Community | 654 | 8-13 |  | 3.34 |
